# Plasma lipidomics profiling identified lipid biomarkers in distinguishing early-stage breast cancer from benign lesions

**DOI:** 10.18632/oncotarget.9124

**Published:** 2016-05-02

**Authors:** Xiaoli Chen, Hankui Chen, Meiyu Dai, Junmei Ai, Yan Li, Brett Mahon, Shengming Dai, Youping Deng

**Affiliations:** ^1^ Department of Clinical Laboratory, The Fourth Hospital Affiliated to Guangxi Medical University, Liuzhou City, Guangxi Province, China; ^2^ Department of Medicine, Rush University Medical Center, Chicago, Illinois, USA; ^3^ Department of Pathology, Rush University Medical Center, Chicago, Illinois, USA; ^4^ Department of Anatomy and Cell Biology, Rush University Medical Center, Chicago, Illinois, USA; ^5^ edical College, Wuhan University of Science and Technology, Wuhan, Hubei Province, China

**Keywords:** breast cancer, lipidomics, benign lesion, biomarker, plasma

## Abstract

**Background:**

Breast cancer is very common and highly fatal in women. Current non-invasive detection methods like mammograms are unsatisfactory. Lipidomics, a promising detection method, may serve as a novel prognostic approach for breast cancer in high-risk patients.

**Results:**

According the predictive model, the combination of 15 lipid species had high diagnostic value. In the training set, sensitivity, specificity, positive predictive value (PPV) and negative predictive value (NPV) of the combination of these 15 lipid species were 83.3%, 92.7%, 89.7%, and 87.9%, respectively. The AUC in the training set was 0.926 (95% CI 0.869-0.982). Similar results were found in the validation set, with the sensitivity, specificity, PPV and NPV at 81.0%, 94.5%, 91.9%, and 86.7%, respectively. The AUC was 0.938 (95% CI 0.889-0.986) in the validation set.

**Methods:**

Using triple quadrupole liquid chromatography electrospray ionization tandem mass spectrometry, this study was to detect global lipid profiling of a total of 194 plasma samples from 84 patients with early-stage breast cancer (stage 0–II) and 110 patients with benign breast disease included in a training set and a validation set. A binary logistic regression was used to build a predictive model for evaluating the lipid species as potential biomarkers in the diagnosis of breast cancer.

**Conclusion:**

The combination of these 15 lipid species as a panel could be used as plasma biomarkers for the diagnosis of breast cancer.

## INTRODUCTION

Breast cancer is the most frequently diagnosed cancer and the second-most leading cause of cancer-related deaths among women in the United States [1]. According to an estimate of the American Cancer Society (ACS) in 2015, 231,840 new breast cancer cases were diagnosed, which accounts for 29% of all newly diagnosed female cancer patients. It is estimated that in the same study that a total of 40,290 breast cancer deaths, amounting to 15% of the cancer-related deaths among women that year [1]. Early diagnosis plays a key role in patients' prognosis. Mammography is currently the most widely used method in breast cancer scanning. However, the outcome is often not satisfactory because of the high false positive rate [2].

The rate of over-diagnosis of breast cancer in screening mammography is wide, from 0% to upwards of 30% [3]. Women with abnormal screening mammograms undergo additional expensive magnetic resonance imaging (MRI) and tissue sampling (by fine-needle aspiration, core biopsy, or excisional biopsy). What makes matters worse is that approximately 10% of women will be called back from each screening examination for further testing, but only 5% of these women will have cancer with the other cases turning out to be benign [4]. Chiarelli et al. [5] had reported that breast MRI plus mammography is an effective method for breast cancer screening. However, it is very expensive and it has not been demonstrated that screening high-risk populations with MRI has translated into a survival benefit [6]. Furthermore, MRI has a high false-positive rate and could lead to a high frequency of futile biopsies, causing additional stress and costs [7]. To avoid unnecessary expensive and invasive screening for those benign patients, a better method is urgently needed. Blood-based tumor markers are one of the research hotspots in the diagnosis of cancers. However, they are not yet used in clinical trials [8–10]. Serum tumor markers such as CA15.3 and BR27.29 are not used for breast cancer detection for their low sensitivity [11]. Thus, there is a pressing need for minimally invasive methods and early diagnosis of malignant breast lesions.

Lipids are involved in regulating many physiological activities, such as energy storage, structure, apoptosis, and signaling [12]. It is reported in many studies that dyslipidemia, as a major component of metabolic syndrome plays an important role in the carcinogenesis of various cancers, including prostate cancer, ovarian cancer and kidney cancer [13–15]. For breast cancer, it has been well documented that metabolomics or lipidomics have shown potential for cancer diagnosis and progression [16–18]. However, most of these studies have just focused on total levels of lipids in cancer patients, and only a few of them included patients with benign breast diseases. Recently, Yang et al. performed a comprehensive evaluation of plasma lipid profiles with benign breast disease patients in only 5 breast cancer cases and 6 benign patients, indicating the diagnostic efficiency of the lipid markers in these diseases [19].

In our study, lipidomics technology and electrospray ionization tandem mass spectrometry (ESI-MS/MS) is employed to conduct a quantitative analysis of plasma samples in both a training set and a validation set with a total of 84 breast cancer patients and 110 benign patients. The whole set (the combined training and validation sets) is used to verify the credibility of the results. In this study, we identified a panel of plasma lipid species which were able to distinguish the early-stage of breast cancer from benign lesions, and serve as potential biomarkers for early diagnosis of breast cancer.

## RESULTS

### Characteristics of patients

A total of 84 patients with early-stage breast cancer (stage 0–II) and 110 with benign breast disease were included in our study. The mean age was 57.7±12.0 Years in the breast cancer group, 47.8±10.9 in the benign group. Among these patients, the breast cancer group had 79 (94%) caucasians and 5 (6%) non-caucasians. In the benign group, there were 103 (94%) caucasians and 7 (6%) non-caucasians. Therefore, most of the patients were caucasians in our study (> 90%). The composition based on stages of breast cancer was as follows: 15 (18%) patients were stage 0, 58 (69%) patients were stage I, and 11 (13%) patients were stage II. According to the samples from different departments, the breast cancer and benign samples were divided into a training set of 90 patients and a validation set of 94 patients. The training and validation set samples were approximately age- and race-matched. The details are shown in Table 1.

**Table 1:** The characteristics of the patients with cancer and benign lesion in the training and validation set

	Rush Training set		CHTN Validation set	
	Cancer (39)	Benign (51)	Cancer (45)	Benign (59)
Gender				
Female	39	51	45	59
Age range (years, mean±SD)	57.5±12.0	59.8±11.1	58.0±12.4	62.1±11.1
Race				
Caucasian	37	49	42	54
Non-caucasian	2	2	3	5
Cancer stage				
0	6		9	
I	27		31	
II	6		5	
Cancer subtypes				
Invasive	33		35	
In situ	6		10	

SD:standard deviation.

### Lipid profiling of lipid species

**Figure 1:**
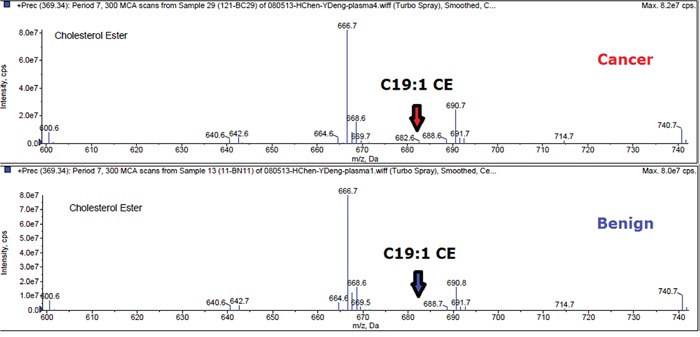
Mass spectra of C19:1 CE in breast cancer and benign

Plasma lipid profiles, including 367 lipid species from 13 classes of phospholipids and 1 class of CE, were identified by lipidomics from a total of 194 plasma samples (84 with breast cancer and 110 with benign breast disease). Due to our test utilizing the method of lipid micro-extraction, a level of lipid species less than 0.0007 nmol/uL was considered likely unreliable. In order to guarantee the quality of lipid species, we removed lipids with more than 40% missing data or outlier mean expression. Accordingly, 367 lipid species were reduced to 191 lipid species. As an example, the mass spectra of C19:1 CE was shown in Figure 1 for a patient with breast cancer and a patient with benign breast disease.

We analyzed the concentration of lipid species from both breast cancer and benign plasma specimens. In the training set, the most significant difference in mean plasma concentration was PC (38:3) (*p* = 2.50297E-08, Student's t-test). The significant fold change was LPC (20:0) (fold-change = 4.08). In the validation set, the most significant difference in mean plasma concentration was PC (38:3) (*p* = 5.70481E-11, Student's t-test). The significant fold change was C 19:0 CE (fold-change = 4.39). In the whole set (the combined training and validation sets), the most significant difference in mean plasma concentration was PC(38:3) (*p*=1.00749E-17, Student's t-test). The significant fold change was C 19:0 CE (fold-change = 3.73). These data indicated that plasma lipid species could be biomarkers for the diagnosis of breast cancer.

### Identification of lipid species as biomarkers for the diagnosis of early-stage breast cancer from benign lesions

**Figure 2:**
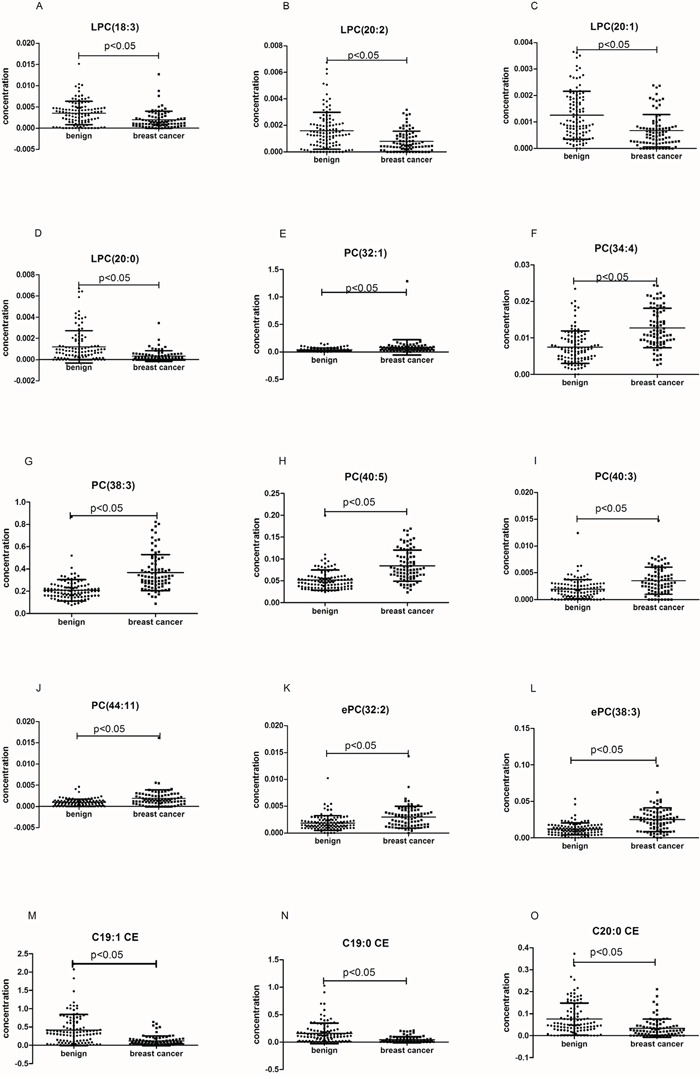
The plasma concentrations of the selected lipid species in the whole set The black horizontal lines are median values. *p* values were determined by the students' T-test.

We analyzed the change in the concentration of 191 lipid species in the training set. The *p* value of the Student's t-test and the fold-change of the average of the concentration of each lipid species were calculated between breast cancer samples and benign samples. According to the filtering condition (p < 0.05 and fold-change > 1.5), only 15 lipid species were selected as biomarkers for the diagnosis of breast cancer (Table 2). The concentration distribution of these selected lipid species is shown in Figure 2. Among these 15 lipid species, there were 4 LPC, 6 PC, 2 ePC, and 3 CE species (Table 2). Compared to that found in benign patients, the plasma concentration of the two classes of LPC and CE were observed to decrease in cancer patients, while the other lipid species increased (Table 2).

**Table 2:** The detection of lipid species as potential biomarkers for diagnosis of early stage breast cancer

Lipid species	Formula	Training set								Validation set							
		P value	Fold- change	SN (%)	SP (%)	PPV (%)	NPV(%)	AUC (95% CI)	Trend (Cancer)	P value	Fold-change	SN (%)	SP (%)	PPV (%)	NPV(%)	AUC (95% CI)	Trend (Cancer)
LPC(18:3)	C26H48O7PN	0.0001	1.73	61.9	70.9	61.9	70.9	0.326(0.213-0.439)	down	0.000725	1.89	61.9	72.7	63.4	71.4	0.314(0.208-0.421)	down
LPC(20:2)	C28H54O7PN	0.000867	2.19	64.3	67.3	60.0	71.2	0.320(0.213-0.426)	down	0.006665	1.84	59.5	69.1	59.5	69.1	0.303(0.198-0.409)	down
LPC(20:1)	C28H56O7PN	0.002848	2.13	64.3	70.9	62.8	72.2	0.269(0.169-0.370)	down	0.02302	1.65	54.8	67.3	56.1	66.1	0.324(0.216-0.431)	down
LPC(20:0)	C28H58O7PN	0.000183	4.08	73.8	65.5	62.0	76.6	0.289(0.186-0.392)	down	0.000719	3.32	66.7	65.5	59.6	72.0	0.306(0.202-0.410)	down
C19:1 CE	C46H84NO2	1.31E-05	3.17	71.4	67.3	62.5	75.5	0.270(0.166-0.374)	down	2.04E-05	3.68	81.0	63.6	63.0	81.4	0.260(0.160-0.360)	down
C19:0 CE	C46H86NO2	0.000285	3.24	71.4	69.1	63.8	76.0	0.286(0.184-0.388)	down	1.12E-06	4.39	78.6	63.6	62.3	79.5	0.262(0.163-0.362)	down
C20:0 CE	C47H88NO2	0.000436	2.09	57.1	74.5	63.2	69.5	0.303(0.196-0.410)	down	0.001025	2.36	64.3	67.3	60.0	71.2	0.292(0.189-0.395)	down
PC(32:1)	C40H78O8PN	4.46E-06	1.97	52.4	83.6	81.0	69.7	0.776(0.680-0.871)	up	0.000942	2.36	38.1	80.0	59.3	62.9	0.723(0.619-0.827)	up
PC(34:4)	C42H76O8PN	3.84E-08	1.84	57.1	85.5	75.0	72.3	0.824(0.740-0.907)	up	9.96E-05	1.57	50.0	80.0	65.6	67.7	0.736(0.636-0.837)	up
PC(38:3)	C46H86O8PN	2.5E-08	1.70	54.8	87.3	76.7	71.6	0.822(0.737-0.908)	up	5.7E-11	1.83	66.7	90.9	84.8	78.1	0.870(0.797-0.942)	up
PC(40:5)	C48H86O8PN	2.92E-06	1.58	50.0	83.6	70.0	68.7	0.765(0.666-0.863)	up	1.27E-09	1.70	64.3	85.5	77.1	75.8	0.839(0.757-0.920)	up
PC(40:3)	C48H90O8PN	9.16E-05	1.88	54.8	85.5	74.2	71.2	0.729(0.624-0.835)	up	0.000657	1.75	42.9	83.6	66.7	65.7	0.670(0.559-0.781)	up
PC(44:11)	C52H82O8PN	0.014073	2.06	45.2	83.6	67.9	66.7	0.716(0.612-0.821)	up	0.000228	2.15	42.9	87.3	72.0	66.7	0.707(0.600-0.815)	up
ePC(32:2)	C40H78O7PN	0.000226	1.60	54.8	89.1	79.3	72.1	0.731(0.625-0.837)	up	0.010426	1.60	42.9	80.0	62.1	64.7	0.655(0.543-0.766)	up
ePC(38:3)	C46H88O7PN	4.32E-05	1.93	61.9	87.3	78.8	75.0	0.765(0.660-0.870)	up	6.69E-06	2.19	61.9	89.1	81.3	75.4	0.754(0.648-0.860)	up
combination	-	-	-	83.3	92.7	89.7	87.9	0.926(0.869-0.982)	-	-	-	81.0	94.5	91.9	86.7	0.938(0.889-0.986)	-

SN:sensitivity; SP: specificity; PPV: positive predictive value; NPV: negative predictive value; AUC: Area under ROC curve.

**Figure 3:**
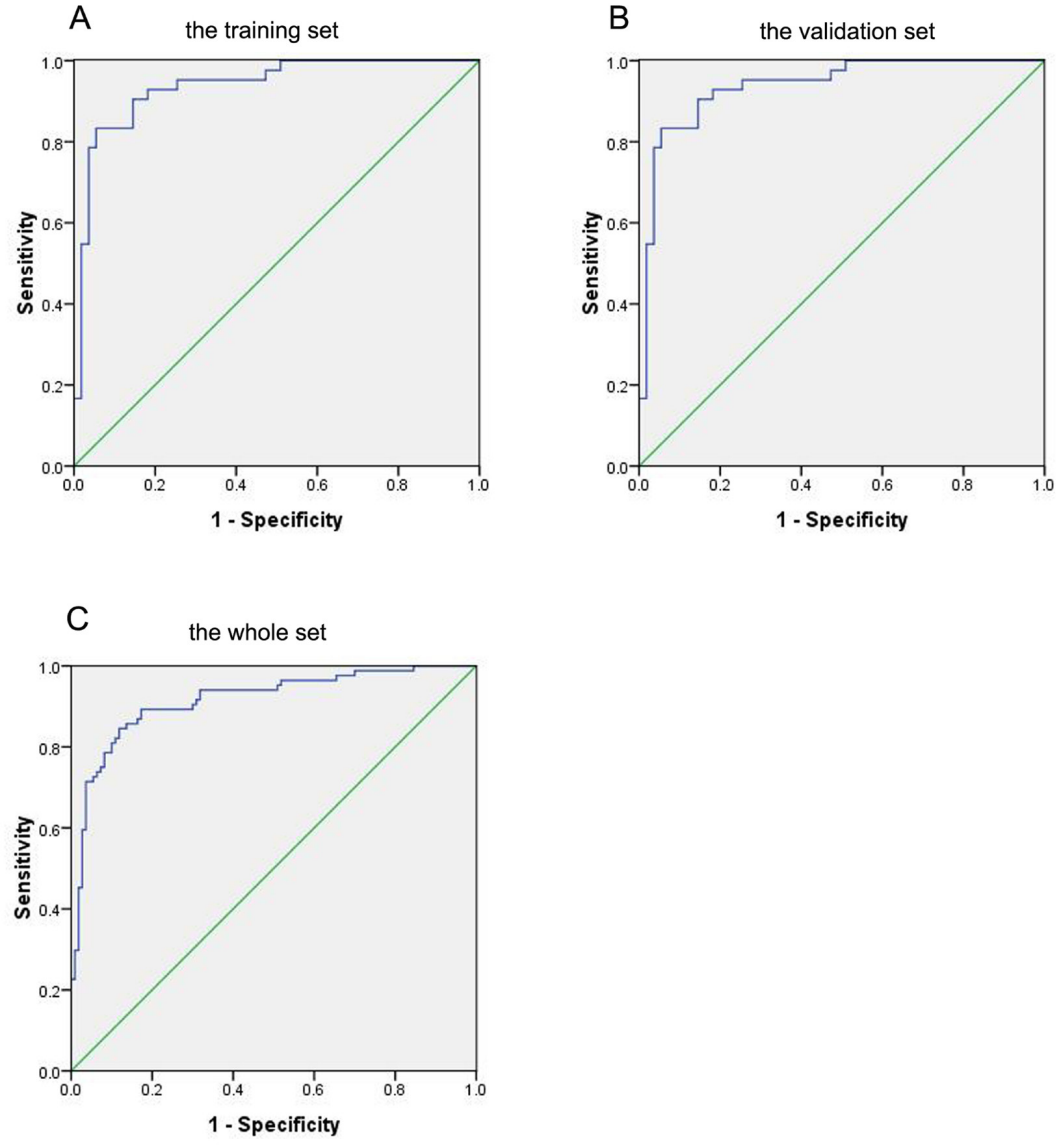
ROC curve of the combination of 15 lipid species in the prediction of breast cancer **A.** Breast cancer versus benign in the training set. **B.** Breast cancer versus benign in the validation set. **C.** Breast cancer versus benign in the whole set.

To test the predictive value of the 15 selected lipids for breast cancer, a binary logistic regression was used to build a predictive model. According to the predictive model, we could further evaluate the performance of the selected lipid species in distinguishing breast cancer patients from benign patients. We found that single lipid species did not have good diagnostic performance in distinguishing breast cancer patients from benign patients. However, the combination of these 15 lipid species had the best diagnostic performance. The sensitivity, specificity, positive predictive value (PPV) and negative predictive value (NPV) of the combination these 15 lipid species were 83.3%, 92.7%, 89.7%, and 87.9%, respectively. The AUC was 0.926 (95% CI 0.869-0.982) (Figure 3A).

In order to further verify these 15 lipid species as potential biomarkers in the diagnosis of breast cancer, we used the same method to analyze the data of the validation set (Table 2). Similar results were found in the validation set. The sensitivity, specificity, PPV and NPV were 81.0%, 94.5%, 91.9%, and 86.7%, respectively. The AUC were 0.938 (95% CI 0.889-0.986) (Figure 3B). In the whole set (the combination of the training set and the validation set), the sensitivity, specificity, PPV and NPV were 81.0%, 90.0%, 86.1%, and 86.1%, respectively (AUC 0.916, 95% CI 0.874-0.957) (Figure 3C).

## DISCUSSION

Breast cancer is very common and highly fatal in women. Mammography is currently used in breast cancer screening, with the sensitivity merely at 54% to 77% [20]. Most abnormal mammograms are false positives that require further investigation including expensive breast imaging and biopsies, which can cause physiological distress. Due to the limitations of mammography, radiological interpretation of indeterminate micro-calcifications as benign or malignant may be unreliable [21]. Thus, a new diagnostic technique with high accuracy for the diagnosis of breast cancer, particularly for distinguishing early cancer from benign lesions, is still needed in clinical practice.

Lipids may be broadly defined as hydrophobic or amphipathic small molecules that originate entirely or in part by carbanion-based condensations of thioesters and/or by carbocation-based condensations of isoprene units [22]. Lipids have been implicated as important roles in several human diseases, including breast cancer [23]. In particular, complex polar lipids may participate in oncologic processes, including breast cancer development and metastasis [16]. In our study, we identified 15 lipid species showing significant differences of plasma concentration between breast cancer and benign patients. The plasma concentrations of PC and ePC classes were revealed to increase in the breast cancer patients, while the others decreased. These results might be caused by the regulation mechanisms of cellular metabolism. PCs, which are known as the major phospholipids found in the membranes of mammalian cells, were mediated by PLA2 in breast cancer cells [24]. Some studies had reported that PLA2 is over-expressed in breast cancer cells [25–27]. The level of the PCs may reflect a higher activity of PLA2. Several articles had shown that different exosomes derived from a variety of cells contained different components, for example heat shock proteins, annexin, lipids, and so on [28–30]. Phuyal S et al. had reported that an increase in cellular ether lipids (including PCs) was associated with changes in the release and composition of exosomes in PC-3 cells [31]. The ePCs belong to subclasses of PCs, and ePCs activate Pl-3-kinase and may participate in mitogenic responses [32]. LPCs and CEs were derived from PCs [33]. The decreased levels of LPCs were associated with an activated inflammatory status in cancer patients [34]. LPCs not only have inflammatory activities, but also activate signaling molecules including tyrosine kinases [35–37]. The binding of LPCs to their receptors may regulate signaling pathways including inflammation and cell migration [35, 38, 39]. The lower levels of LPCs may reflect a higher metabolism rate in breast cancer patients. The metabolic effect of CE in breast cancer remains poorly understood. But the relationship between CE and poor clinical outcome in human breast cancer has been reported [40]. These studies have indicated that these selected lipid species could be classified as biomarkers for the diagnosis of breast cancer.

Our current data showed that a single plasma lipid species was unlikely to perform well in distinguishing breast cancer from benign patients. However, the combination of the selected lipid species had a high diagnostic value for breast cancer prediction with high sensitivity, specificity, PPV, NPV and AUC, as shown in the training set, the validation set, and the whole set. Furthermore, the specificity of the combination of 15 selected lipid species for breast cancer (the training set: 92.7%, the validation set: 94.5%) was higher than mammograms, suggesting that these lipid markers could be potential biomarkers for the diagnosis of breast cancer among women with abnormal mammograms.

As far as we know, this is the first study on plasma lipid biomarkers in distinguishing early-stage breast cancer from benign lesions in a large sample set. Our aim is to identify circulating lipid signatures that can be used reliably as a companion diagnostic tool together with screening mammography, to reduce the number of unnecessary follow-up investigations, especially invasive biopsy. Using a triple quadrupole LC-ESI-MS/MS, the lipid profiling was able to achieve fast, high-efficiency and high-throughput detection. The test only required 3uL of plasma, which involved only a minimally invasive procedure. After biostatistical analysis, a highly sensitive and specific predictive model was developed for the diagnosis of breast cancer. The cost of the detection of global lipid profiling is high. However, measurement of a panel of 3-15 plasma lipid species may be feasible in clinical laboratories. For this reason, the selected lipid species were used as diagnostic biomarkers only, but not as screening biomarkers.

There were some limitations in our study. First, the benign group included many benign diseases, such as hyperplasia, fibroadenomas, cysts and some unspecified findings diagnosed in this organ. According to the small sample size for each benign disease, we could not conduct a subgroup analysis. Second, most of the patients in our study were caucasian (> 90%). Third, due to incomplete information related to the tumor size, we were unable to conduct a correlational analysis between the lipid species and tumor size. Therefore, the diagnostic performance of lipid species in breast cancer still needs to be confirmed by further studies with rigorous design, more cooperation, and larger sample sizes.

## MATERIALS AND METHODS

### Patients and plasma samples collection

The training cohort included 39 Breast cancer and 51 benign samples, which were obtained from the Rush Breast Cancer Repository. The patients were selected according to the following criteria: (1) all patients were diagnosed and confirmed by pathology; (2) patients with breast cancer were at the early stages (stage 0, I, II) according the clinical staging method; (3) patients had no other diseases which might affect lipid metabolism such as hyperlipidemia, diabetes, and other cancers; (4) all patients were female; and (5) none of the patients received preoperative adjuvant chemotherapy or radiotherapy. Breast benign lesions are defined as hyperplasia, fibroadenomas, cysts and some unspecified findings diagnosed in this organ. Control blood samples were collected from healthy women with no history of malignant diseases and no inflammatory conditions.

According to these criteria, we also collected plasma samples from 45 patients with early-stage breast cancer (stage 0–II) and 59 patients with benign breast disease from the Cooperative Human Tissue Network (CHTN) Western Division and Southern Division. All cancer patient histopathology results were confirmed by surgical resection of the tumors, while clinicohistopathological characteristics and tumor stage were assessed based on histobiopsy results. No preoperative chemotherapy or radiotherapy was applied to cancer patients included in this study. All of these cancer, benign and control samples were approximately age- and race-matched, as shown in Table 1. Rush University Medical Center IRB approved on study, with written consent for the use all the subject information and biospecimens.

Before the collection of plasma samples, patients fasted at least 12 hours. Briefly, for plasma isolation, blood was collected into Vacutainer tubes with EDTA (BD, Franklin Lakes, NJ) and centrifuged at 2,600g for 10 minutes at 4°C within 2 hours of venipuncture. The supernatant was removed and centrifuged in the same way for the second time. Plasma was stored in 0.5 mL aliquots at −80°C. All plasma samples were transported to the Kansas Lipidomics Research Center (KLRC) for lipid analysis with dry ice.

### LC-ESI-MS/MS lipid profiling

According to the method of Bligh and Dyer [41], the lipids were extracted from the plasma with some modifications. 3μL of plasma was used for each sample analysis. Each sample was centrifuged at 10,000 rpm for 20 minutes at room temperature on a table tube unit to pellet the proteins prior to detection. In order to obtain exact identification of all lipid species, precise amounts of internal standards were added. Two internal standards were used for each class of lipid species. After centrifuging, the lipid extracts were re-dissolved in the solvents for HPLC injection. The solvents were the rate of chloroform/methanol/300mM ammonium acetate in water (μL) was 360/840/44. All solvents used were HPLC grade.

Lipid profiling was performed by a triple quadrupole LC-ESI-MS/MS (API 4000, Applied Biosystems, Foster City, CA), which was based on collision-induced dissociation (CID) for structural identification. The sample introduction is continuous injection of electrospray ionization (ESI) saurce. It could reduce the ionization suppression effect caused by spectral congestion [42]. ESI of complex lipids generates singly charged ions that can produce fragments by CID. With the help of LC-ESI-MS/MS, lipids can be distinguished by their polar heads and their chain lengths.

Lipid data acquisition was carried out as described previously [43–46]. Two types of scans were used to obtain polar lipid profiles: precursor and neutral loss scans. Lipid species in a class are identified as precursors of, or as ions that undergo neutral loss of, a common head group fragment. A custom script and Applied Biosystems Analyst software were used for the resolution of chromatographic peaks. After mass filtering, alignment, and internal standard normalization, the data were quantified in the unit of nmol/μL.

### Statistics analysis

SPSS 17.0 software was used for statistical analyses. The differences between the two plasma sample sets were evaluated by the Student's t-test. All *p* values were derived from two-sided test. Differences were considered statistically significant when *p* values were less than 0.05 and fold-change was larger than 1.5.

Further statistical analysis was performed with SPSS software. According to the binary logical regression analysis, we could predict the diagnostic efficiency of the selected lipid species. The “Enter” method was chosen to estimate the diagnostic accuracy of lipid. Receiver operating characteristic (ROC) curves were plotted to assess the relation of sensitivity and specificity. Area under ROC curve (AUC) with 95 % confidence interval (CI) was also calculated. Scatter plots were generated by GraphPad Prism version 5 for Windows.

## CONCLUSION

This study assessed the combination of lipid species as a panel for the diagnosis of breast cancer. Our findings indicate that a procedure using biostatistical analysis on a lipid profile is capable of producing a highly sensitive and specifically predictive model that classifies patients between having benign and malignant breast cancer. These results show that lipid profiles may be a promising avenue for the investigation of diagnostic biomarkers of breast cancer.

## Abbrevation

LPC: lysophosphatidylcholine
PC: phosphatidylcholine
ePC: ether-linked phosphatidylcholine
CE: cholesterol ester
PLA2: phospholipase A2
